# Performance evaluation of the RITG148^+^ set of TomoTherapy quality assurance tools using RTQA^2^ radiochromic film

**DOI:** 10.1120/jacmp.v17i4.6178

**Published:** 2016-07-08

**Authors:** Eric C. Lobb

**Affiliations:** ^1^ Department of Radiation Oncology St. Elizabeth Hospital Appleton WI USA

**Keywords:** tomotherapy, quality assurance, RIT, automation, radiochromic film

## Abstract

Version 6.3 of the ${\text{RITG}148}^{+}$ software package offers eight automated analysis routines for quality assurance of the TomoTherapy platform. A performance evaluation of each routine was performed in order to compare ${\text{RITG}148}^{+}$ results with traditionally accepted analysis techniques and verify that simulated changes in machine parameters are correctly identified by the software. Reference films were exposed according to AAPM TG‐148 methodology for each routine and the ${\text{RITG}148}^{+}$ results were compared with either alternative software analysis techniques or manual analysis techniques in order to assess baseline agreement. Changes in machine performance were simulated through translational and rotational adjustments to subsequently irradiated films, and these films were analyzed to verify that the applied changes were accurately detected by each of the ${\text{RITG}148}^{+}$ routines. For the Hounsfield unit routine, an assessment of the “Frame Averaging” functionality and the effects of phantom roll on the routine results are presented. All ${\text{RITG}148}^{+}$ routines reported acceptable baseline results consistent with alternative analysis techniques, with 9 of the 11 baseline test results showing agreement of 0.1 mm/0.1° or better. Simulated changes were correctly identified by the ${\text{RITG}148}^{+}$ routines within approximately 0.2 mm/0.2° with the exception of the Field Center vs. Jaw Setting routine, which was found to have limited accuracy in cases where field centers were not aligned for all jaw settings due to inaccurate autorotation of the film during analysis. The performance of the ${\text{RITG}148}^{+}$ software package was found to be acceptable for introduction into our clinical environment as an automated alternative to traditional analysis techniques for routine TomoTherapy quality assurance testing.

PACS number(s): 87.55.Qr

## I. INTRODUCTION

Radiological Imaging Technology, Inc. (Colorado Springs, CO) is a software manufacturer that provides tools for automated radiotherapy quality assurance and diagnostic imaging quality control test analysis. Recently the ${\text{RITG}148}^{+}$ package was introduced into the RIT113 version 6.3 product family and marketed as a comprehensive suite of quality assurance test tools for the TomoTherapy platform (Accuray, Sunnyvale, CA).^(1)^ These tools were designed to facilitate quantitative and automated analysis for a number of quality assurance tests recommended in the Report of the AAPM Task Group (TG) 148 titled “QA for helical tomotherapy.”^(2)^

At the time of writing there are seven available test modules: “Beam Planarity and Jaw Twist,” “Overhead Laser Positioning,” “MLC Center of Rotation,” “Field Center vs. Jaw Setting,” “Couch Translation Per Gantry Rotation,” “Static Gantry Angle Tool,” and “Helical Gantry Angle Tool”. Additionally, there is support for automated Hounsfield unit (HU) vs. electron and physical density analysis within the Image QA module using CT images of the TomoTherapy “Cheese” phantom (Gammex RMI, Middleton, WI). Though not evaluated here, Version 6.4 of ${\text{RITG}148}^{+}$ includes additional testing routines for completion of interrupted procedures, verification of alignment between the imaging system and treatment beam, and additional imaging tests for noise, uniformity, contrast, and resolution.^(3)^

With the exception of the HU analysis test — which is recommended to be performed monthly when TomoTherapy MVCT imaging is used for dose calculations — these tools correspond to a subset of the recommended TG‐148 quarterly and annual quality assurance tests. Additionally, these tests are recommended following replacement or servicing of TomoTherapy components such as the MLC hardware and jaw assembly. Measurements for these tests are commonly acquired using radiographic or radiochromic film and are analyzed either by hand, with the TomoTherapy Film Analyzer software (Accuray Inc., Madison, WI), or using custom in‐house solutions.

The ${\text{RITG}148}^{+}$ software tools can serve as an alternative to these analysis techniques provided they report accurate results and are able to identify changes in machine parameters or errors in hardware alignment following servicing. This study was designed to systematically investigate the performance of this suite of TomoTherapy quality assurance tools, including their agreement with other independent analysis techniques and their ability to accurately detect and report simulated changes in machine performance for each test.

## II. METHODS

Each tool in this suite underwent a testing process that began with an initial reference measurement following the TG‐148 methodology in order to establish RIT‐reported baseline performance values that subsequent measurements could be compared against. These measurements were also analyzed using independently established techniques that are used for routine quality assurance analysis in our department in order to evaluate baseline consistency. The TomoTherapy Film Analyzer software (version 1.1.2.6) was used for independent analysis of measurements for tests where it had a corresponding routine. Tests that do not have a TomoTherapy Film Analyzer routine were independently analyzed using manual profile‐based analysis in accordance with departmental quality assurance protocols. Descriptions of the reference measurement techniques are provided in each respective section below. The reader is referred to the AAPM TG‐148 report and its references for information related to the purpose and motivation for each of these quality assurance tests.

Measurements were performed using Gafchromic RTQA2 radiochromic film (Ashland Specialty Ingredients, Bridgewater, NJ), which is a reduced‐cost film designed for routine quality assurance applications. All films originated from the same batch and were handled with tissue paper to prevent fingerprint artifacts. Exposed films were digitized as reflective photo documents with a 48‐bit color depth and 96 dots‐per‐inch resolution using an Epson Expression 10000 XL flatbed scanner (Epson America Inc., Long Beach, CA) with no color corrections or scanning filters. All films for a specific routine were exposed in the same session and digitized approximately 1 hour after exposure.

Simulation of changes in machine performance was accomplished using three techniques: translational motion of the TomoTherapy couch, rotation of the film on a rotating base, and direct modification of procedure delivery parameters. The specific technique utilized for each test is discussed in the following routine‐specific sections.

The TomoTherapy Hi‐Performance Couch can be step‐adjusted in the lateral (IEC‐X), longitudinal (IEC‐Y), and vertical (IEC‐Z) directions in increments of 0.1 mm with a manufacturer‐stated point‐to‐point accuracy of better than 0.5 mm. This study utilizes translational couch motions of 0.2–6.0 mm, requiring verification of acceptable couch performance at these levels. Couch motion relative to the fixed overhead laser system was tested using a ruler with 0.5 mm gradations. Step‐adjustments in the IEC‐X and IEC‐Y directions of 0.5, 1.0, 2.0, 3.0, and 6.0 mm were verified directly to be accurate to within better than 0.125 mm through comparison of programmed couch travel and motion of the overhead laser along the ruler length. Step‐adjustments of 0.2 mm were verified to be accurate through a process of applying a 20 mm couch adjustment in increments of 0.2 mm. The total physical travel of the couch as measured on the ruler differed by less than 0.125 mm from the programmed 20 mm travel.

**Figure 1:**
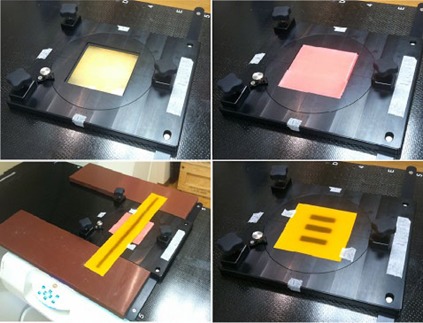
Top left shows the rotating base with 1.3 cm superflab bolus material used to fill the central void; top right, the 0.2 cm dental wax layer used for completing the buildup/backscatter layer; bottom left, the rotating base setup when using 43.2 cm long film sheets; bottom right, the rotating base setup when using 12.7 cm square film sheets.

The rotating base consisted of a 22 cm diameter plastic plate with angle markings in increments of 1° and a central void filled with near‐tissue‐equivalent buildup and backscatter materials (Fig. 1). The plate was set into a rectangular leveling platform that can be indexed to the TomoTherapy couch. The 1° markings were verified to be spaced appropriately through comparison against both a handheld protractor and the rotating crosshair projection in the light field of a Varian Truebeam (Varian Medical Systems, Inc., Palo Alto, CA) linear accelerator. Additional markings were made on the rotating platform at an angle of ±0.5° using the Varian Truebeam crosshair. A rotation of 0.25°±0.125° was achieved through rotating the plate until the reference mark was visually centered between the 0° and 0.5° markings.

For films placed in the coronal plane, orientation markings were made that designated the direction of gantry‐right as seen from the foot of the couch. For films placed in the transverse plane, the orientation markings designated gantry‐right and were made to point approximately toward the entrance direction of a 45° beam. Additional markings were made as needed for each routine according to the ${\text{RITG}148}^{+}$ software manual. During analysis within the RIT software, these markings were digitally erased and replaced with a 3‐pixel by 3‐pixel digital pinprick centered on the physical location of the markings.

Results presented in this study were initially acquired under Version 6.3 of the RIT113 software and subsequently validated under Version 6.4.

### A. Beam planarity and jaw twist tool

The Beam Planarity and Jaw Twist tool verifies the perpendicular alignment of the treatment beam transverse central axis defined by the Y jaw against the rotational plane of the gantry, as well as whether or not the Y jaw is parallel to the gantry's rotation plane. In this test, a sheet of film was placed between 1.5 cm of buildup material with the center of the film aligned laterally (IEC‐X) and longitudinally (IEC‐Y) with machine isocenter. The couch was lowered to its most extreme position and the vertical (IEC‐Z) distance between the film and machine isocenter was measured as 25.0 cm ($d_{\text{film}}$). The film was exposed for 70 s with the gantry at 0° and MLC leaves 1–32 open using the 1 cm jaw setting. A second exposure of 40 s was made with the gantry rotated to 180° and all other beam parameters remaining the same.

The Beam Planarity and Jaw Twist tool reports six quantities based on the exposed film and input of the $d_{\text{film}}$ value: 1) mean jaw offset projected to isocenter (IEC‐Y), 2) jaw twist angle, 3) median jaw offset projected to isocenter (IEC‐Y), 4) jaw offset maximum, 5) jaw offset minimum, and 6) jaw offset standard deviation. Quantities 1) and 2) are of interest in this study, with TG‐148 recommended tolerances of ±0.5 mm and ±0.5°, respectively.

Jaw offset changes were simulated by applying IEC‐Y couch shifts between the two film exposures. The jaw offset projected to isocenter is related to the offset measured on film and the quantity $d_{\text{film}}$ by Eq. (1).

(1) $$Offset_{ISO}=Offset_{FILM}\times \left[\frac{85cm}{2\times d_{film}}\right]$$

Jaw shifts projected to isocenter of 0.34, 0.51, and 1.02 mm were simulated by shifting the couch in the IEC‐Y direction by 0.2, 0.3, and 0.6 mm, respectively.

Jaw twist changes were simulated by rotating the film between the 0° and 180° exposures. Care was taken during this step to ensure that the center of rotation coincided with the beam central axis and that the junction of the two exposure areas was not translated in the IEC‐X or IEC‐Y directions. Because the physical jaw twist is half of the angle between the two exposure regions on the film, jaw twists of 0.25°, 0.5°, and 1.0° were simulated with film rotations of 0.5°, 1.0°, and 2.0°, respectively.

### B. Overhead laser position tool

The Overhead Laser Position tool is used to verify the alignment between the overhead fixed laser and the treatment beam. There are three results reported by this analysis routine: 1) longitudinal offset from the center of the exposure to the transverse laser line (IEC‐Y), 2) rotation of the transverse laser line with respect to the exposure, and 3) lateral offset from the center of the exposure to the sagittal laser line (IEC‐X). Translational alignment should be within 1 mm with a maximum rotation of 0.3°, as recommended by TG‐148.

The analysis requires two points be marked on the film along the transverse laser line, one each to the right and left of the exposure region. The sagittal alignment result is available only if two additional points are marked along the sagittal laser line in the positive and negative IEC‐Y direction relative to the exposure region.

The reference exposure was acquired using a 43.2 cm‐wide sheet of film positioned at isocenter with 1 cm of solid water buildup. The transverse and sagittal lasers were marked as required with no shifts introduced. The exposure lasted 60 seconds and utilized the 1 cm jaw width with all MLC leaves open.

Changes in the longitudinal alignment of the laser system were simulated by marking the film along the transverse laser line and then adjusting the IEC‐Y position of the couch by 0.5 mm,

1.0 mm, and 2.0 mm. Alignment changes for the sagittal laser were separately simulated using IEC‐X couch adjustments of 0.5 mm, 1.0 mm, and 2.0 mm. Rotational changes were introduced by rotating a marked film by 0.25°, 0.5°, and 1.0° with no translational shifts.

Prior to these measurements the TomoTherapy laser system underwent a full calibration and alignment check. These checks verified that the translational alignment of the lasers with virtual isocenter was correct within 0.3 mm, that the rotational alignment of the lasers with the treatment beam was correct within 0.3°, and that each laser was plumb or level, as appropriate, within better than 0.5 mm across a 50 cm distance.

### C. MLC twist and center of rotation tool

The MLC Twist and Center of Rotation tool is used to verify the lateral alignment of the MLC hardware with respect to the gantry center of rotation, along with verification that the MLC hardware is parallel to the plane of rotation. This routine reports five quantities: 1) MLC offset, 2) MLC twist, 3) center‐to‐center distance between the right and central exposure regions, 4) center‐to‐center distance between the left and central exposure regions, and 5) largest percent area difference between exposure regions. Quantities 1) and 2) are of interest in this study with TG‐148 tolerances of 1.5 mm and 0.5°, respectively.

When following the testing methodology described in AAPM TG‐148 the MLC offset and twist measured on film at isocenter is a factor of 2 greater than the physical offset and twist of the MLC hardware. The results reported by the ${\text{RITG}148}^{+}$ tool are the physical offset and twist of the hardware, not the uncorrected on‐film results. The reference exposure for this test was acquired using a film positioned at isocenter between two 1.5 cm thicknesses of buildup. The film was exposed for 60 s from gantry angle 0° with MLC leaves 27, 28, 32, and 33 open. The film was then exposed for an additional 60 s from gantry angle 180° with only MLC leaves 27 and 28 open.

Lateral offset changes were simulated through IEC‐X couch adjustments between the 0° and 180° exposures. Changes of 0.5, 1.0, 2.0, and 3.0 mm were simulated through couch adjustments of 1.0, 2.0, 4.0, and 6.0 mm, respectively. Adjustments to MLC twist of 0.25°, 0.5°, and 1.0° were simulated by rotating the film between exposures by 0.5°, 1.0°, and 2.0°, respectively.

### D. Field center vs. jaw setting tool

The Field Center vs. Jaw Setting tool verifies that the beam axis for each of the fixed Y‐jaw settings (1.0, 2.5, and 5.0 cm) share a common center within a TG‐148‐recommended tolerance of 0.5 mm. The tool for this test locates the IEC‐Y center of seven adjacent exposure regions and reports the individual coordinate results, as well as the maximum difference between any two field centers. Any combinations of field sizes may be used for this test so the user may compare either two or all three field sizes with a single film. The routine attempts to autoalign the image without the need for markings on the film.

For the reference exposure, the film was exposed using three consecutive procedures (with no interprocedure couch motion) in order to include all of the jaw settings on a single film. The film was placed at isocenter under 1.5 cm of solid water and each exposure was 80 s in duration. The MLC pattern for the 1.0 cm exposure opened leaves 2–9, 20–27, and 47–54. The MLC pattern for the 2.5 cm exposure opened leaves 11–18, 38–45, and 56–63. The MLC pattern for the 5.0 cm exposure opened leaves 29–36 only.

The introduction of simulated jaw‐centering errors required only an IEC‐Y adjustment of the couch position between exposures. Two films were exposed that included shifts in the 1.0cm and 2.5 cm procedures relative to the 5.0 cm procedure. For the 1.0 cm jaw setting, these shifts were 0.5 mm and 1.0 mm, while for the 2.5 cm jaw these shifts were 0.2 mm and 2.0 mm. The analysis consisted of recording the separation between each field center relative to the 5.0 cm exposure for the reference measurement, and then rerecording this data for each exposure on the two films with shifts applied. The resultant change in separation between the central exposure and any of the other six exposures should ideally match the applied shift for that particular film and field size.

### E. Couch translation per gantry rotation tool

The Couch Translation per Gantry Rotation tool verifies the synchronization between the rotating gantry and the translating couch during delivery of a helical procedure. The film exposure was performed using a user‐created delivery sinogram that opens the full MLC bank at predefined intervals during the procedure to create three lines of exposure spaced 50 mm apart with a TG‐148 tolerance of 1 mm. If the position and speed of the rotating gantry is out of sync with the translational motion of the couch, these lines will appear either too close together or too far apart.

The reference procedure utilized the 1.0 cm field width with a pitch value of 1.0. There were 13 rotations planned with all MLC leaves opening at gantry angles between 270° and 90° during rotations 2, 7, and 12. For this procedure the film was placed at the IEC‐X and IEC‐Z isocenter location under 1.5 cm of solid water. Changes in exposure spacing were generated by modifying the delivery procedure to have a pitch of 0.99, 0.98, and 0.96 in order to produce lines of exposure spaced at 49.5, 49.0, and 48.0 mm, corresponding to expected changes of 0.5, 1.0, and 2.0 mm, respectively.

### F. Static gantry angle tool

The Static Gantry Angle tool verifies the accuracy of the gantry positional calibration by comparing lines of exposure against film markings placed along the IEC‐X and IEC‐Z axes. Meaningful test results rely on accurate marking of these coordinate axes and so an appropriate technique must be used for generating these references. A properly calibrated gantry‐facing laser system will project level and orthogonal lasers along the IEC‐X and IEC‐Z axes. Prior to this test, it was verified that the gantry‐facing lasers were plumb and level to within less than 0.5 mm across 50 cm of lateral and vertical distance in the plane of machine isocenter. Accurate markings on the film along these laser projections therefore are plumb and level to within better than 0.06°.

This routine requires four exposure lines be generated, generally expected to start at 0° and progress in 45° increments to 135°. The angle of each of the four detected lines of exposure is reported relative to the IEC‐Z axis. For the reference exposure a film was positioned at isocenter parallel to the treatment plane between two 4 cm slabs of solid water. The reference gantry angles were 0°, 45°, 90°, and 135° and the procedures were delivered without interprocedure couch motion. Changes were simulated for this test by programming the static exposures to be made at angles of 0.2°, 45.5°, 91°, and 137° degrees, corresponding to gantry position offsets of 0.2°, 0.5°, 1.0°, and 2.0°, respectively.

### G. Helical gantry angle tool

The Helical Gantry Angle tool tests the ability of the TomoTherapy platform to correctly reproduce programmed gantry angles during a helical delivery in which film exposures are made near the beginning and end of the procedure using two sequentially irradiated films separated by a user‐defined distance (typically 5–6 cm). In each rotation, the central MLC leaves open only for a small number of projections centered at angles 0°, 120°, and 240°, creating thin lines of exposure that can be compared against reference level markings on the film to determine the accuracy of the gantry position calibration. The reference markings are placed along the IEC‐X and IEC‐Z axes in the same way as for the previously discussed Static Gantry Angle tool. The gantry rotates a set number of times as the couch translates longitudinally, resulting in two films with their own “helical star shot” pattern that can be independently analyzed.

The analysis routine for this tool does not produce results until two films have been imported, corresponding to the “front” and “back” films. For each film the routine provides the measured gantry angle for each of the three lines of exposure relative to the IEC‐Z axis.

As with the static gantry angle tool discussed above, simulated changes in gantry calibration were introduced by modifying the control sinogram to open the leaves at offset intervals. In this case the sinogram was modified to open the leaves at 0.5°, 121°, and 242°, corresponding to changes of 0.5°, 1.0°, and 2.0°.

### H. Image QA (Hounsfield unit tool)

The RIT Image QA module has a “Cheese Phantom” routine that overlays circular regions of interest (ROI) onto an image slice of the Gammex “Cheese” phantom in order to perform automated evaluation of the HU values of up to 20 materials. The names of the materials can be specified through modification of the “ritDiagPrefs” configuration file, while the order and relative electron/physical density information can be directly modified from within the tool preferences.

The size of the ROI used for measuring the mean HU values can be set to between 5 and 25 mm in diameter. The diameter of a typical Gammex density plug is 27.5 mm, though specialty plugs containing higher atomic‐number rods (such as stainless steel or titanium) embedded in solid‐water cylinders may have material diameters of 12.5 mm or less.

The Image QA tool also allows for frame‐averaging of 3, 5, or 7 DICOM images, which may be useful for improving measurement statistics when using small ROIs for plugs with smaller diameters. The effect of the various frame‐averaging techniques on the mean HU values and the standard deviation of the result was evaluated in comparison to a single‐frame technique to assess the potential benefit of its utilization.

The ROIs for the density plugs are autoplaced by the software based on the known geometry of the phantom. Misalignments of the phantom vertically or laterally are corrected automatically by the routine prior to ROI placement, and this functionality was tested for offsets of up to 10 mm. Rotational errors in the phantom setup are not corrected or reported by the RIT software. When using ROI diameters that are of a similar size to the density plugs in the image, small rotations may move parts of the material rods outside of the ROI and result in artificially low or high HU values. An evaluation of the effect of phantom rotations on automated HU value analysis is presented for rotations of 1°‐5°, applied electronically using the ImageJ software.^(4)^

## III. RESULTS AND DISCUSSION

A summary of agreement results between RIT and the Film Analyzer software or manual analysis techniques for baseline measurements is presented in Table 1. All routines reported baseline results within 0.2 mm/0.2° of independent analysis methods, with 9 of 11 routines showing agreement of better than 0.1 mm/0.1°, demonstrating acceptable baseline consistency between the ${\text{RITG}148}^{+}$ software and independent analysis techniques.

Simulated errors were correctly identified for all tests within better than approximately 0.2° and 0.2 mm for the following routines: Beam Planarity and Jaw Twist (Table 2), MLC Twist and Center of Rotation (Table 3), Couch Translation per Gantry Rotation (Table 4), Static/Helical Gantry Angle (Table 5), and Overhead Laser Position (Table 6). Routine‐specific results and discussion for the remaining routines are presented in their own sections.

**Table 1:** Summary of agreement between RIT‐reported results and results from independent analysis techniques for baseline measurements

*Summary of Baseline Results*			
	*RIT Reported Result*	*Independent Result*	*Agreement (Absolute)*
Beam Planarity (mm)	‐0.18	‐0.16 ^a^	0.02
Jaw Twist (mm)	0.02	‐0.03 ^a^	0.05
Overhead Laser IEC‐Y (mm)	‐0.25	‐0.21 ^a^	0.04
Overhead Laser IEC‐X (mm)	0.00	0.08^a^	0.08
Overhead Laser Rotation (degrees)	‐0.03	‐0.01 ^a^	0.02
MLC IEC‐X Offset (mm)	1.04	1.20^a^	0.16
MLC Rotation (degrees)	0.00	0.00^b^	0.00
Field Center vs. Jaw Setting (mm)	0.17	0.17^b^	0.00
Couch Translation/Gantry Rotation Mean(mm)	49.83	49.83^b^	0.00
	0.00	0.08^b^	0.08
Static Gantry Angle (degrees)	45.08	45.17^b^	0.09
	89.98	90.00^b^	0.02
	134.96	134.81^b^	0.15
	‐0.04	‐0.11 ^b^	0.07
Helical Gantry Angle (degrees)	119.90	119.92^b^	0.02
	240.10	240.16^b^	0.06

a ^a^ Results were acquired using the TomoTherapy Film Analyzer software.

b ^b^ Results were acquired using manual analysis techniques.

**Table 2:** Results for testing of the Beam Planarity and Jaw Twist tool

*Beam Planarity and Jaw Twist Tool*			
	*Applied Offset*	*Measured Offset*	*Change from Baseline*	*Agreement*
	0.00 (Baseline)	‐0.18	‐	‐
Beam Planarity (mm)	0.34 0.55	0.16 0.39	0.34 0.57	0.00 0.02
	1.02	0.78	0.96	‐0.06
	0.00 (Baseline)	0.02	‐	‐
Jaw Twist (degrees)	0.25 0.50	0.11 0.48	0.09 0.46	‐0.16 ‐0.04
	1.00	0.98	0.96	‐0.04

**Table 3:** Results for testing of the MLC Center of Rotation tool

*MLC Twist and Center of Rotation Tool*				
	*Applied Offset*	*Measured Offset*	*Change from Baseline*	*Agreement*
	0.00 (Baseline)	1.04	‐	‐
	0.50	1.56	0.52	0.02
IEC‐X (mm)	1.00	2.10	1.06	0.06
	2.00	3.09	2.05	0.05
	3.00	4.17	3.13	0.13
	0.00 (Baseline)	0.00	‐	‐
Rotation (degrees)	0.25	0.15	0.15	‐0.10
	0.50	0.58	0.58	0.08
	1.00	1.04	1.04	0.04

**Table 4:** Results for testing of the Couch Translation per Gantry Rotation tool. In the third and fourth columns, respectively, “1 vs. 2” and “2 vs. 3” refer to the interexposure spacing between the center of exposures 1, 2, and 3. Each spacing delta has two data points, and so the mean values are presented in the final two columns. All units are in millimeters

*Couch Translation Per Gantry Rotation Tool*					
		*Measured*	*Measured*		
*Applied Spacing*	*Applied Spacing* Δ	*Spacing (1 vs. 2)*	*Spacing (2 vs. 3)*	*Mean Change from Baseline*	*Mean Agreement*
50.00 (Baseline)	0.00	49.80	49.85	‐	‐
49.50	‐0.50	49.40	49.34	‐0.45	0.05
49.00	‐1.00	48.99	48.83	‐0.91	0.09
48.00	‐2.00	47.90	47.74	‐2.01	0.00

**Table 5:** Results for the Static and Helical Gantry Angle tools

Static Gantry Angle Tool/Helical Gantry Angle Tool					
	*Set Angle*	*Angle Offset*	*Measured Angle*	*Change from Baseline*	*Agreement*
Static Gantry (degrees)	0.00 (Baseline)	‐	0.00	‐	‐
	45.00 (Baseline)	‐	45.08	‐	‐
	90.00 (Baseline)	‐	89.98	‐	‐
	135.00 (Baseline) 0.20	0.20	134.96 0.34	0.34	0.14
	45.50	0.50	45.80	0.72	0.22
	91.00	1.00	91.09	1.11	0.11
	137.00	2.00	137.01	2.05	0.05
Helical Gantry (degrees)	0.00 (Baseline)	‐	‐0.04	‐	‐
	120.00 (Baseline)	‐	119.90	‐	‐
	240.00 (Baseline) 0.50	0.50	240.10	‐	‐
	0.50	0.50	0.46	0.50	0.00
	121.00	1.00	120.93	1.03	0.03
	242.00	2.00	242.10	2.00	0.00

**Table 6:** Results for testing of the Overhead Laser Position tool

*Overhead Laser Position Tool*					
	*Applied Offset*	*Measured Offset*	*Change from Baseline*	*Agreement*
IEC‐Y (mm)	0.00 (Baseline)	‐0.25	‐	‐
	0.50	0.20	0.45	‐0.05
	1.00	0.82	1.07	0.07
	‐2.00	‐2.04	‐1.79	0.21
IEC‐X (mm)	0.00 (Baseline)	0.00	‐	‐
‐0.50	‐0.50	‐0.50	0.00
1.00	1.19	1.19	0.19
2.00	2.21	2.21	0.21
Rotational (degrees)	0.00 (Baseline)	‐0.03	‐	‐
‐0.25	‐0.17	‐0.14	0.11
0.50	0.49	0.52	0.02
1.00	1.00	1.03	0.03

### A. Field center vs. jaw setting tool

Results of the Field Center vs. Jaw Setting tool are provided in Table 7. Shifts in IEC‐Y positioning of various exposure regions were in general not identified by the ${\text{RITG}148}^{+}$ routine consistently with the physical shifts. Results corresponding to the 0.20 and 1.00 mm shifts applied to the first test film were found to vary widely by exposure region, up to a maximum absolute discrepancy of approximately 0.6 mm for the furthest left and right exposures. Similarly for the 0.50 and 2.00 mm shifts applied to the second test film, maximum discrepancies of approximately 1.3 mm were found for the furthest left and right exposures.

**Figure 2:**
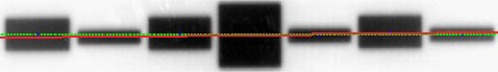
Discrepancy between autorotated orientation and manually rotated orientation of “Film 2” with the Field Center vs. Jaw Setting tool. The autorotation level line is shown in green, while the pinprick‐based level line using the overhead laser system is shown in red. The difference in rotational alignment is approximately 0.5°.

These discrepancies are likely the result of improper autorotation of the film by the ${\text{RITG}148}^{+}$ routine prior to analysis (Fig. 2). When comparing the orientation of the autorotated films in the RIT routine to the orientation of the same films when leveling was performed according to pinpricks along the overhead laser projection, a discrepancy of approximately 0.3° and 0.5° was seen for the first and second test films, respectively. When performing a manual field‐centering analysis of the films after laser‐based leveling the maximum discrepancy between anticipated and measured results across the 12 measurements improved to ‐0.16 mm, with 9 of the 12 results decreasing to less than a 0.1 mm discrepancy.

**Table 7:** Results for testing of the Field Center vs. Jaw Setting tool. The rows labeled “RIT Test” show results for automated RIT analysis using the RIT‐determined film orientation. Rows labeled “Manual Test” show results for profile‐based manual analysis using film orientation based on the overhead laser system

*Field Center vs. Jaw Setting Tool*						
	*Exposure Region*	*Baseline IEC‐Y Position*	*Applied IEC‐Y Shift*	*Measured Shifted IEC‐Y Position*	*Change from Baseline*	*Agreement*
RIT Test 1 (mm)	1 (1.0 cm)	0.02	1.00	0.08	0.40	‐0.60
	2 (2.5 cm)	0.03	‐0.20	‐0.94	‐0.63	‐0.43
	3 (1.0 cm)	‐0.04	1.00	0.47	0.85	‐0.15
	4 (5.0 cm)	0.02	0.00	‐0.32	‐	‐
	5 (2.5 cm)	‐0.06	‐0.20	‐0.39	0.01	0.21
	6 (1.0 cm)	0.10	1.00	1.10	1.34	0.34
	7 (2.5 cm)	‐0.07	‐0.20	‐0.02	0.39	0.59
RIT Test 2 (mm)	1 (1.0 cm)	0.02	0.50	‐0.09	‐0.76	‐1.26
	2 (2.5 cm)	0.03	‐2.00	‐2.18	‐2.86	‐0.86
	3 (1.0 cm)	‐0.04	0.50	0.75	0.14	‐0.36
	4 (5.0 cm)	0.02	0.00	0.67	‐	‐
	5 (2.5 cm)	‐0.06	‐2.00	‐0.97	‐1.56	0.44
	6 (1.0 cm)	0.10	0.50	1.95	1.20	0.70
	7 (2.5 cm)	‐0.07	‐2.00	‐0.12	‐0.70	1.30
Manual Test 1 (mm)	1 (1.0 cm)	32.125	‐1.00	32.288	‐0.98	0.02
	2 (2.5 cm)	32.125	0.20	33.484	0.21	0.01
	3 (1.0 cm)	32.167	‐1.00	32.331	‐0.98	0.02
	4 (5.0 cm)	32.167	0.00	33.312	‐	‐
	5 (2.5 cm)	32.250	0.20	33.612	0.22	0.02
	6 (1.0 cm)	32.250	‐1.00	32.331	‐1.07	‐0.07
	7 (2.5 cm)	32.292	0.20	33.654	0.22	0.02
Manual Test 2 (mm)	1 (1.0 cm)	32.125	‐0.50	31.818	‐0.54	‐0.04
	2 (2.5 cm)	32.125	2.00	34.349	1.99	‐0.01
	3 (1.0 cm)	32.167	‐0.50	31.776	‐0.62	‐0.12
	4 (5.0 cm)	32.167	0.00	32.398	‐	‐
	5 (2.5 cm)	32.250	2.00	34.390	1.91	‐0.09
	6 (1.0 cm)	32.250	‐0.50	31.817	‐0.66	‐0.16
	7 (2.5 cm)	32.292	2.00	34.390	1.87	‐0.13

For the baseline test where the field center for each jaw setting was properly aligned, the ${\text{RITG}148}^{+}$ routine reported results that were in exact agreement with manual analysis techniques. When field centers are not properly aligned the autorotation of the film performed by the routine may limit the accuracy of the reported results. This could be remedied by allowing the user to perform pinprick‐based leveling using the overhead lasers.

It should be noted that, for both test films, the reported maximum difference between the centers of any two exposures was roughly a factor of 2 greater than the physical result due to these film rotation issues. Because the reported difference between exposure centers is exaggerated by the ${\text{RITG}148}^{+}$ routine, it is not likely that this autorotation issue would lead to a passing result being reported for an out‐of‐tolerance set of exposures.

### B. Image QA (Hounsfield unit tool)

**Figure 3:**
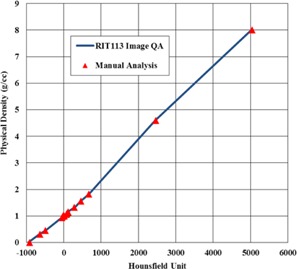
Comparison of MVCT physical density conversion data between the RIT113 image QA routine and manual analysis using ImageJ.

When using an 8 mm diameter circular ROI setting with a centered and level CT image the Image QA tool reports HU values for each density plug consistently with manual techniques using ImageJ, indicating appropriate autoplacement of the plug‐specific ROIs (Fig. 3).

**Figure 4:**
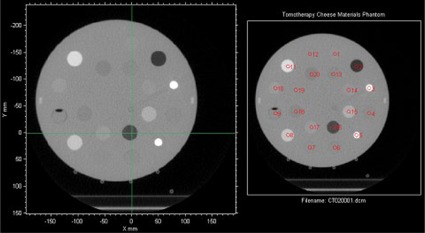
Resultant 8 mm ROI placement (red circles in the right image) for a phantom image that is grossly offset, both vertically and laterally, from the center of the imaging field of view (green crosshair in the left image). ROI placement is appropriate despite offset phantom positioning.

**Figure 5:**
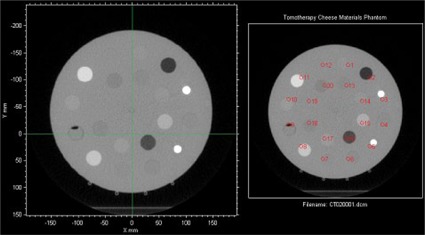
Resultant 8 mm ROI placement (red circles in right image) for a phantom that has been physically rotated approximately 10°. ROI positioning is offset from the density plugs and will yield incorrect HU values.

The RIT software appropriately corrected for translational offsets in the phantom position within the imaging field of view prior to placement of ROIs (Fig. 4) with no significant difference in HU values for any IEC‐X or IEC‐Z phantom offsets. However, the introduction of rotational offsets resulted in improper placement of ROIs on the phantom image that was not automatically corrected by the software (Fig. 5).

**Figure 6:**
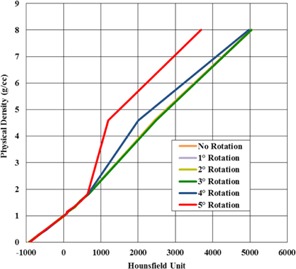
Comparison of MVCT physical density conversion data for a number of phantom rotations. Data for rotations of 0°‐3° is indistinguishable when plotted.

Results of phantom rotations on HU values are presented qualitatively in Fig. 6 for an 8 mm ROI diameter. When applying phantom rotations of 1°‐3° there was no discernable effect on the mean HU results for each plug. A rotation of 4° or more caused the ROI for the smallest 12.5 mm plugs to move partly into the surrounding solid water, leading to artificially low HU results. Mean HU values for the larger 27.5 mm plugs were not influenced by phantom rotations up to even 5° when using an 8 mm ROI. If using only the larger 27.5 mm plugs, the ROI diameter can be set up to a maximum of 16 mm while preserving robustness against phantom rotations up to 3°, based on displacement of a density plug placed in the outermost ring of the “Cheese” phantom. Users should be able to achieve phantom rotations of 0.3° or less without difficulty, so this should not be a prominent concern in most cases. Nonetheless, users utilizing this routine for initial generation of density calibration curves should be aware that unintentionally rotated phantoms could result in subtly erroneous data if there is no baseline for comparison.

Results of frame‐averaging tests are presented in Table 8 for both 12 mm and 27.5 mm density plugs. Across all frame‐averaging techniques the mean HU value for both density plugs varied by approximately 0.5%. For both density plugs, the 7‐frame‐averaging technique provided a one‐sigma standard deviation (SD) that was approximately half of its value when no frame averaging was used. Frame averaging can speed up the process for clinicians who choose to average mean HU values from multiple slices when generating a CT calibration curve and can result in a reduction in uncertainty for the data points, but the absolute HU values are unlikely to be significantly affected.

**Table 8:** Results for frame‐averaging tests using the Image QA “Cheese Phantom” routine

*Frame‐Averaging Results on HU Measurement*			
*Density Plug Size*	*Frames Averaged*	*Mean HU Value*	*SD (1σ)*
12.0 mm Diameter	1 (Single‐Frame)	2501.03	39.99
12.0 mm Diameter	3	2485.32	27.03
12.0 mm Diameter	5	2493.06	23.03
12.0 mm Diameter	7	2486.85	21.81
27.5 mm Diameter	1 (Single‐Frame)	675.44	26.78
27.5 mm Diameter	3	677.65	18.77
27.5 mm Diameter	5	677.97	15.76
27.5 mm Diameter	7	678.80	13.76

## V. CONCLUSIONS

The ${\text{RITG}148}^{+}$ set of TomoTherapy analysis routines in RIT version 6.3 provides automated analysis for seven mechanical and synchronicity‐based quality assurance tests and one imaging quality assurance test. The Image QA tool accomplishes its function of providing automated HU value analysis for the “Cheese” phantom, and is robust against translational positioning errors and phantom rotations up to 3° when using appropriately sized regions of interest. For film‐based routines, the RIT‐reported results were satisfactorily consistent with baselines established with other accepted analysis techniques. When introducing simulated machine changes into each routine near the level of each test's tolerance, the change in RIT‐reported results was consistent with the expected changes for all routines, except for the Field Center vs. Jaw Setting tool.

For the Field Centering tool, the accuracy of the reported results for misaligned field centers appears limited by the autorotation applied to the input film, but this inaccuracy leads to reported field center offsets that are larger than the physical offsets and would, therefore, still appropriately alert the medical physicist that the test has failed. The accuracy of this routine could be improved by expanding the options for rotational alignment of the film to include manual input of rotation correction or through rotational corrections based on film landmarks.

Overall, the performance of the ${\text{RITG}148}^{+}$ routines was found to be acceptable for introduction into the author's clinical environment for ongoing quality assurance testing. Readers who wish to perform their own performance analysis prior to utilization of these tools can use these results as a comparative example against their own findings.

## COPYRIGHT

This work is licensed under a Creative Commons Attribution 3.0 Unported License.
